# Can infrared light really be doing what we claim it is doing? Infrared light penetration principles, practices, and limitations

**DOI:** 10.3389/fneur.2024.1398894

**Published:** 2024-08-28

**Authors:** Theodore A. Henderson

**Affiliations:** ^1^Neuro-Luminance, Inc., Denver, CO, United States; ^2^Neuro-Laser Foundation, Denver, CO, United States; ^3^Dr. Theodore Henderson, Inc., Denver, CO, United States; ^4^The Synaptic Space, Inc., Denver, CO, United States; ^5^The International Society of Applied Neuroimaging (ISAN), Toronto, ON, Canada

**Keywords:** transcranial, traumatic brain injury, NIR, photobiomodulation, NILT, penetration, class IV laser, brain

## Abstract

Near infrared (NIR) light has been shown to provide beneficial treatment of traumatic brain injury (TBI) and other neurological problems. This concept has spawned a plethora of commercial entities and practitioners utilizing panels of light emitting diodes (LEDs) and promising to treat patients with TBI and other disorders, who are desperate for some treatment for their untreatable conditions. Unfortunately, an LED intended to deliver photonic energy to the human brain does not necessarily do what an LED pointed at a mouse brain does. There is a problem of scale. Extensive prior research has shown that infrared light from a 0.5-watt LED will not penetrate the scalp and skull of a human. Both the properties of NIR light and the manner in which it interacts with tissue are examined. Based on these principles, the shortcomings of current approaches to treating neurological disorders with NIR light are explored. Claims of clinical benefit from low-level LED-based devices are explored and the proof of concept challenged. To date, that proof is thin with marginal benefits which are largely transient. Extensive research has shown fluence at the level of the target tissue which falls within the range of 0.9 J/cm^2^ to 15 J/cm^2^ is most effective in activating the biological processes at the cellular level which underlie direct photobiomodulation. If low-level infrared light from LED devices is not penetrating the scalp and skull, then these devices certainly are not delivering that level of fluence to the neurons of the subjacent brain. Alternative mechanisms, such as remote photobiomodulation, which may underlie the small and transient benefits for TBI symptoms reported for low-power LED-based NIR studies are presented. Actionable recommendations for the field are offered.

## Introduction

**Figure 1:**
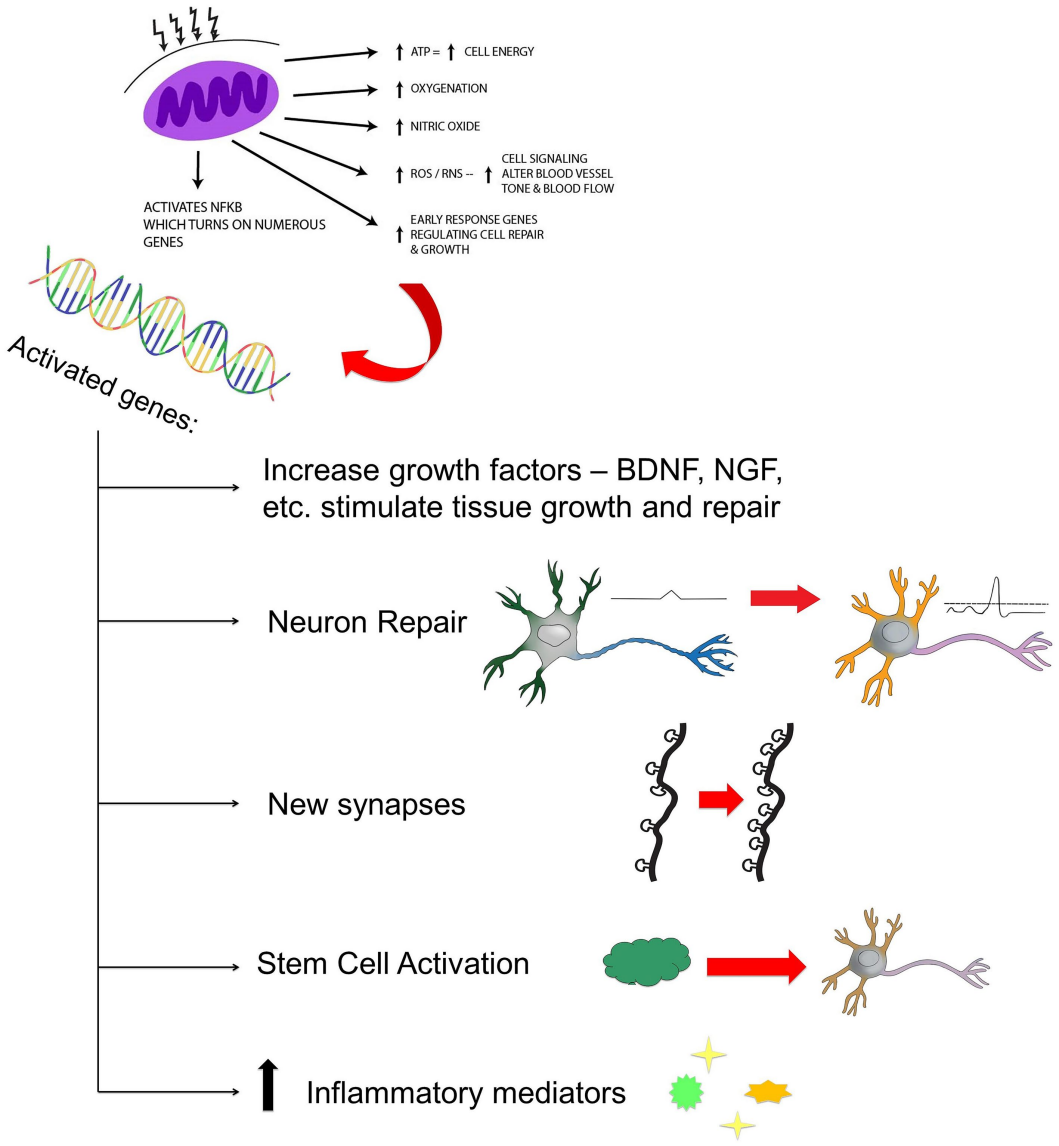
Hypothesized mechanism of action of NIR light therapy. Near infrared light (600–980 nm) penetrates tissue to variable depth depending on wavelength, the tissue involved, coherence, power, and time. A fraction of the photonic energy reaches the mitochondria and is absorbed by cytochrome C oxidase. This activates increased adenosine triphosphate (ATP) production, increased production of reactive oxygen species (ROS), reactive nitrogen species (RNS), and possibly increased nitric oxide. Downstream events include increased early response genes – cfos, cjun and activation of nuclear factor kappa B (NF-kB) - which in turn induces increased transcription of gene products leading to synaptogenesis, neuronal repair, neurogenesis, and increased production of inflammatory mediators and growth factors.

Since entering the field of photobiomodulation over 10 years ago, I have been suspicious of the ability of low-power infrared light to directly activate biological processes in the brain. Light within a fluence range of 0.9 J/cm^2^ to 15 J/cm^2^ and of certain wavelengths is most effective in activating the biological processes at the cellular level which underlie direct photobiomodulation (1–6). Specifically, wavelengths in the range of 600–1,200 nm interact with the mitochondrial electron transport protein, cytochrome c oxidase (COX). As a result, electrochemical potential and ATP production are increased. In addition, multiple secondary and tertiary events occur, likely set in motion by a change in mitochondrial permeability transition pore. Transcription factors (e.g., redox factor-1-dependent activator protein; hypoxia-inducible factor alpha) are upregulated, nitric oxide is displaced from the COX molecule – further stimulating the electron transport chain, and reactive oxygen species are produced. Nuclear factor kappa-B is activated by reactive oxygen species and this leads to the expression of multiple genes which influence apoptosis, inflammation, and neuroplasticity. Further downstream, growth factors are produced leading to increased synaptogenesis, dendritic arborization, and neuroplasticity (see Figure 1).

To be clear, this is not the fluence range delivered to the surface of the skin to initiate photobiomodulation of deep tissues. Rather, this is the fluence range which has penetrated to the level of the target tissue wherein mechanisms within the mitochondria of the target cells are activated. The amount of energy delivered to the surface of the skin must be considerably greater in order to allow for energy loss due to the physical barriers to light penetration represented by the intervening tissues. In terms of treating the brain, there are a number of barriers between the skin surface and the brain, including the tissue of the brain itself. Hence, low level light therapy (LLLT) may fall short of its goal of reaching the brain. While multiple commercial products are emerging with claims of treating the brain directly using low-power light-emitting diodes (LEDs), both the physical properties of light and principles of logic make it impossible for these low-power devices to be *directly treating* the brain. Hence a timely and critical review of the physical properties involved in the interaction of light with tissue is sorely needed to enlighten the photobiomodulation field before it becomes overrun by false claims, misconceptions, and, potentially, charlatans.

In 2019, the late Dr. Larry Morries and I published a chapter in the textbook, *Photobiomodulation in the Brain*, which detailed the physics of infrared light penetration through skin and other tissues (7). We have advocated for a careful look at what the field thinks and claims transcranial infrared light therapies are actually doing since our first paper on the topic in 2015 (4). This article looks at practices and underlying policies in the field of transcranial PBM. It includes an abbreviated summary of the body of work on the penetration of infrared light through tissues with additional review of more recently published data. It will conclude with thoughts, discussion, and actionable recommendations for the field. I fully acknowledge that a portion of the text and figures are adapted from that earlier work (7). Herein, I ask the fundamental question – can infrared light from low-power devices, also known as LLLT, penetrate human scalp and skull, penetrate into the brain, and reach the target neurons therein?

## Parameters of light

Light is a form of electromagnetic radiation with properties of both waves and particles. Light is characterized by its energy, wavelength (distance between two peaks), frequency, and amplitude. Energy is quantified as Joules (J). The amount of energy delivered per unit time constitutes the power of light in Watts (W = J/s). I concur with Jenkins and Carroll (8), who asserts that studies of photobiomodulation should always report wavelength (nm), energy (J), irradiance or power density (W/cm^2^), and radiant exposure [fluence or dose (J/cm^2^)].

As previously mentioned, light in the range of 600–1,200 nm has significant photobiomodulation potential (9). Currently, it is believed that the absorption of infrared photons by the transmembrane protein, COX, in the inner mitochondrial membrane is the key initiating event for photobiomodulation (2, 4, 9–11). COX contains two copper (Cu) centers and two heme-iron centers. Each metal center has a different light absorption peak. Reduction of CuA occurs with 620 nm light, while oxidation of CuA occurs with 825 nm light. Similarly, reduction of CuB occurs with 760 nm light, while oxidation of CuB occurs at 680 nm (9). These peaks correlate with the “optical windows” associated with the biological effects of infrared light (4). Photobiomodulation studies have largely focused on this range of wavelengths. For example, 665 nm, 670 nm, and 810 nm induced neurological and behavioral recovery from TBI in mice (12, 13), while 980 nm light did not. Numerous studies have shown that 810 nm energy induces neurological recovery (1, 12, 14–16), behavioral recovery (1), reduction in lesion volume (1, 15, 16), and induction of the growth factor, brain-derived neurotrophic factor (BDNF) (16). These are believed to result from the direct effect of that particular wavelength on the COX molecule.

## Penetration of infrared light in models of treating human brain

The NeuroThera Effectiveness and Safety (NEST) trials represent the largest and first clinical trials of photobiomodulation in neurological applications. The NEST trials examined clinical efficacy in the treatment of acute stroke of NIR laser light of 808 nm (energy density = 0.9 J/cm^2^) applied to multiple sites on the human scalp for a total of 40 min (17–19). While NEST-1 and NEST-2 showed clinical efficacy for the treatment of acute stroke, NEST-3 failed to reach clinical efficacy, in part, because the inclusion criteria were expanded to include deeper strokes (20). Lapchak (20) asserted that the light energy failed to reach adequate depth with adequate energy in patients with acute stroke to affect a clinically detectable change. This failure to penetrate with adequate fluence is supported by other models of penetration.

In a model of infrared light penetrating scalp, skull, and brain to 3 cm, we have previously reported (4) that infrared light below 10 W simply could not penetrate these same tissues. We utilized lamb heads as a model. We compared the penetration of light from various emitters, including a 50 mW LED emitter of 810 nm light, a commercially produced array of 200 mW LEDs emitting 650 nm and 880 nm light, a commercially available 6 W laser emitting 670 nm and 970 nm light, a commercially available 10 W laser emitting 810 nm and 980 nm light, a Diowave (West Palm Beach, Fl, USA) 15 W laser emitting 810 nm light, and a Diowave 15 W laser emitting 980 nm light. A profound drop in light energy was found when measuring penetration through scalp, skull and 3 cm into the brain. No energy from the 50 mW LED or the 200 mW LEDs could be detected at the depth of 3 cm. Over 99.99% of the energy from the 6 W laser was absorbed by the scalp, skull, and brain tissue. Very small increments of the 10 W and 15 W laser emitters’ energy reached the depth of 3 cm. Specifically, 0.35% of the energy from the 10 W 810/980 nm laser and 2.9% of the energy from the 15 W 810 nm laser penetrated the 3 cm sequence of tissues (4).

In a model of infrared light penetration through a similar thickness of living skin, bone, and connective tissue (2.5 cm thick human hand), Jagdeo et al. (21) found that 0.01–0.09% of impinging infrared light from an LED source (approximately 35 mW/cm^2^ emission 830 nm, not fully defined by the authors) could penetrate 2.5 cm. Similarly, my late colleague, Dr. Morries, and I examined the penetration of infrared light through the living human hand. We found that only 0.6% of infrared light from a 13.5 W laser emitting 810 nm light could penetrate through 2.5 cm of human hand. We also noted that penetration was greater through tissue containing bone – we observed that only 0.3% of the photonic energy from the same source penetrated 2.5 cm of living skin and triceps muscle (4).

If I take the data from Jagdeo and colleagues on the human hand as a model of human scalp, skull, and brain, apply the upper end of their data (0.09% from a 0.5 W LED), and ignore the blocking properties of hair (to be explored later), then we have 0.00045 W (or J/s) penetrating to a 2.5 cm depth through scalp, skull, and brain. If we further assume that this energy falls upon a 1 square centimeter, then that works out to 0.00045 J/cm^2^. In order to reach the level of fluence shown to induce biological processes by direct photobiomodulation, then a time factor of 2,000–33,333 s would need to be applied. This works out to an exposure time of between 33.3 min to 9.263 h. Given that virtually all the clinical studies reported to date, which show clinical benefit, utilized exposure times measured in minutes, not hours, it is difficult to reconcile the claims of clinical benefit of low-power infrared light devices and/or LLLT with the physics of infrared light.

## Interactions with specific tissues

Herein, I will clarify the physical interactions of infrared light with each of the tissues through which it passes en route to the brain. I shall highlight research on light penetration and the clinical studies of traumatic brain injury (TBI) in an effort to illuminate this conundrum.

### Skin

The penetration of infrared light photonic energy through tissues is determined by several factors: wavelength, energy, scatter, absorption, refraction, coherence, area of irradiance, and pulsing. These factors have been explored in detail in previous publications (4, 7, 22). Perhaps the largest contributor in determining the penetration of infrared light to reach the human brain is the scattering properties of the tissues which lie in between the infrared light source and the target tissue. The effects of a given tissue on scattering can be expressed as a scattering coefficient (μ_s_), which is equal to the fraction of light energy dispersed from a light beam per cm. The greater the scattering coefficient, the greater the proportion of light which is scattered over the distance of 1 cm of tissue. Scattering coefficients of selected tissues are shown in Table 1 (7). For example, skin has a scattering coefficient of 46.0. In other words, when NIR photons pass through the skin, approximately half of the photons are scattered. Of course, this increases with thicker skin, such as the scalp (5.5 mm) vs. the average skin (2 mm) (7, 23, 24).

**Table 1:** Scattering coefficient of different tissues [derived from data presented in (7)].

	Mean scattering coefficient (μ_s_)	SD	*N*
Skin	46.0	13.7	8
Bone	22.9	14.6	3
Brain	24.2	11.7	8
Other soft tissues	18.9	10.2	18
Other fibrous tissues	27.1	5.0	5
Fatty tissue	18.4	9.0	6
Breast	16.8	8.1	8

**Figure 2:**
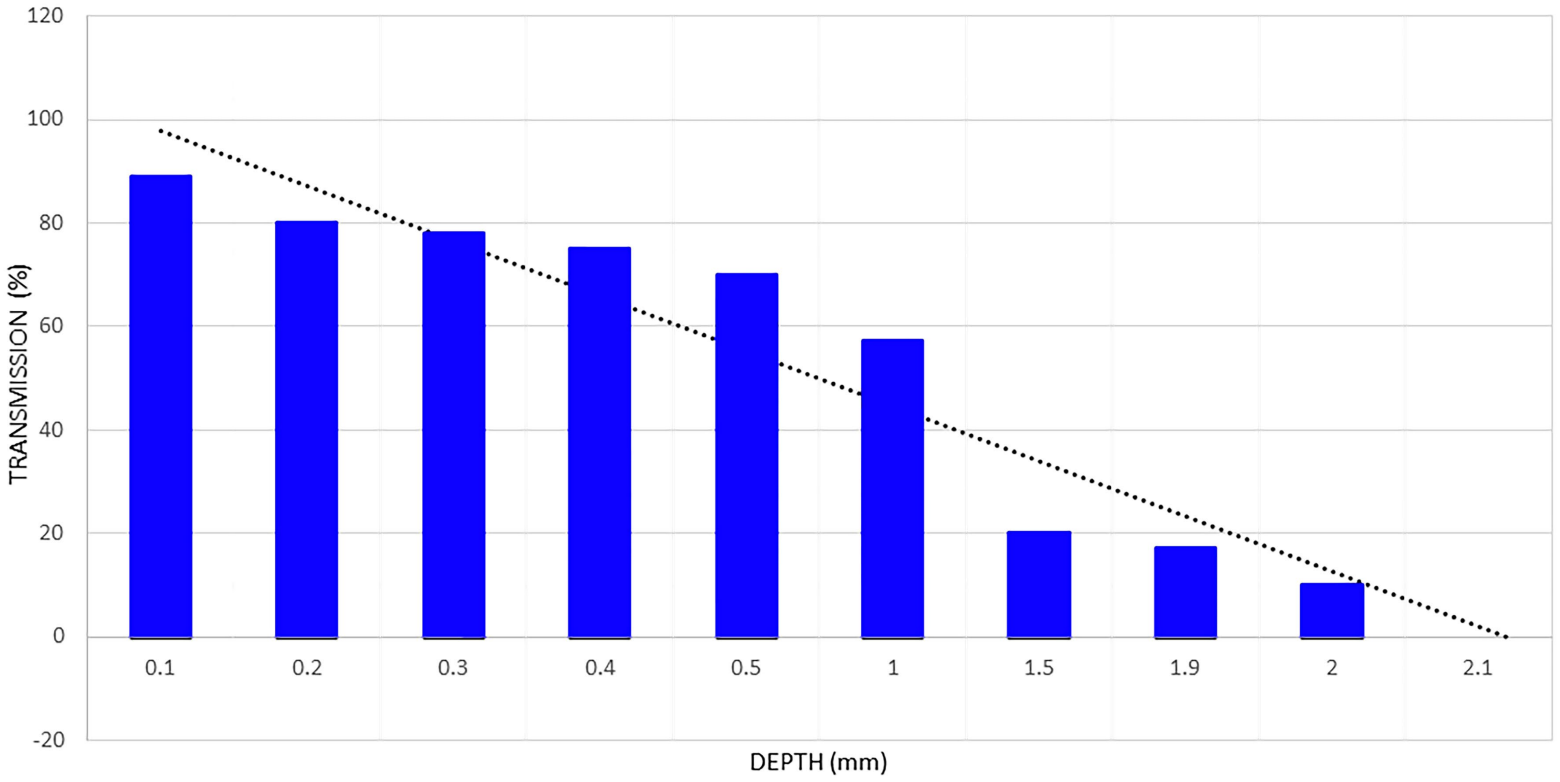
Penetration of light from an 820 nm gallium-aluminum-arsenium laser diode through a sample of fresh human skin. Data extrapolated from data presented in Kolari (25) and shown in the blue columns. A line of regression is shown by the black dotted line. The regression line indicates that light from a low-power laser diode can penetrate less than 2.2 mm into human skin. Based on a figure originally presented in (7).

Kolari illustrated the effects of skin thickness in an early study of how 820 nm low-power infrared laser penetrated through progressively thicker sections of human skin (25). Approximately 78% of 820 nm infrared light penetrated through 0.4 mm of epidermis, but penetration dropped to only 58% at 1 mm (see Figure 2). With 2 mm thick skin, the transmittance of energy dropped to approximately 10% of the incident 820 nm light (Figure 2). At 3 mm 0% of the incident light was detected. These data suggest that skin alone is sufficient to absorb, scatter, or refract all of the photonic energy from a low-power infrared light emitter.

**Figure 3:**
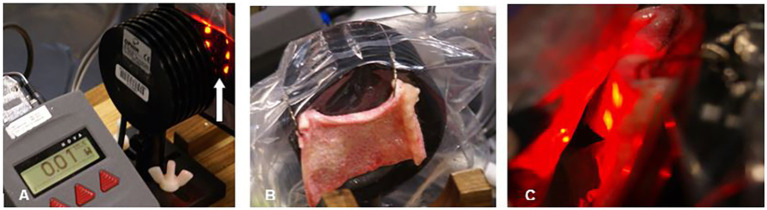
Penetration studies of *ex vivo* human skin illustrated. **(A)** The pad of LED is placed 2 mm from the surface of the light meter detector. The arrow indicates a row of NIR LED with a wavelength of 880 nm. The meter reads 0.1 W. **(B)** Human skin 1.9 mm thick is interposed between the NIR LED and the light meter detector. Thin plastic wrap covers the detector. **(C)** The NIR LED is covered with thin plastic wrap and placed directly against the sample of human skin. Photonic energy could not be detected passing through 1.9 mm of human skin. Originally presented in Henderson and Morries (4).

Esnouf et al. (27) also examined the interaction of infrared light with human skin using an 850 nm continuous infrared light source at 0.10 W. They reported 34% of incident light could penetrate 0.784 mm. Aulahk et al. (28) described a similar experiment using 808 nm laser and found a 0.5 W laser could only penetrate to 6 mm. Dr. Morries and I previously examined infrared light penetration through fresh, unpreserved human skin and fresh, unpreserved sheep skin using several different NIR light sources (4). We showed NIR energy from a 50 mW 810 nm LED did not penetrate 2 mm of human or sheep skin (Figure 3). Similarly, we showed no NIR energy from a commercially available 200 mW LED (650 nm + 880 nm) could be detected penetrating either human skin or sheep skin. In contrast, 9% of the energy from a 10 W 810 nm continuous wave infrared laser passed through 2 mm of sheep skin, while 11% passed through 1.9 mm of human skin. Higher power yielded greater penetration – with a 15 W laser emitting 810 nm light in continuous mode, we found 33% of its energy penetrated through 2 mm of sheep skin, while 17% penetrated 1.9 mm of human skin. We also found that 14% of the energy from a 15 W laser emitting 980 nm light could penetrate 2 mm of sheep skin. We found that pulsing the infrared light improved penetration through skin. For example, 41% of the energy from a 10 W infrared laser emitting 810 nm light with a pulse frequency of 10 Hz penetrated 1.9 mm of human skin, in contrast to only 11% continuous wave light as described above. Similarly, pulsing a 15 W 810 nm laser at 10 Hz delivered 69% of the energy through 1.9 mm of human skin, while only 17% of the continuous wave light of similar parameters could penetrate the same skin sample (4). Morse et al. (29) replicated our study and extended observation to skin from an African American donor. However, they utilized 1 mm thick samples of skin. Approximately 50% of the light from a 1 W source of 750 nm or 940 nm infrared light penetrated approximately 1 mm of human skin. This matches the data from Kolari (25) and our data (see also Figure 2). Notably, there was very little effect of skin pigmentation.

Taken together, these data match the predictions from the theoretical and physical parameters of light interactions with skin. The penetration of low-power infrared light sources below 6 W is limited to the first 3 mm, at most, of human skin. However, human scalp is generally thicker than 3 mm. Scalp thickness in balding and non-balding males and in females has been examined in at least three studies (30–32). Scalp thickness varied from 2.9 to 7.6 mm across studies. Light (32) described a sample of 28 cadaver scalps with an average thickness of 5.1 mm. Garn et al. examined 523 healthy adults and found an average scalp thickness of 5.8 mm. Hori et al. (31) examined 44 cadaver scalps and found thicknesses ranging from 3.3–4.7 mm. They also measured scalp thickness in 19 healthy adults and found thicknesses ranging from 3.9–4.3 mm.

Now, the skin of the forehead overlying a portion of the frontal lobes is approximately 2 mm thick. It is possible that tiny amounts of infrared light from lower powered emitters could penetrate the forehead skin; however, only 9–11% of the light from a 10 W emitter penetrated that thickness of skin. Nevertheless, the remainder of the scalp, over which hoods, helmets, and posteriorly placed LED pads are emitting low-power light, is an average of 5.1–5.8 mm thick.

Simply put, it does not matter how long an LED is shone on a human head if the light energy from that LED cannot penetrate through human skin further than 3 mm. The energy of low-power devices simply will not penetrate the thickness of the scalp overlying much of the skull. Some have suggested that NIR energy from low-power devices penetrates deeper if longer exposure times are used. This reflects a fundamental misunderstanding of the roles that scatter, absorption, and refraction play in degrading NIR energy as it passes through tissue. The energy delivered to the skin surface is different from the energy that penetrates to the depth of the target tissue – often several cm below the surface. Longer exposure times will simply pump more energy into the epidermis and dermis of the skin/scalp. Longer exposure times do not yield deeper penetration. These limitations on penetration only take into consideration the skin and scalp; however, the skull is a formidable barrier to light penetration, as well.

### Skull

The scattering coefficient of bone is considerably lower and averages approximately 22.9 (see Table 1). Fewer photons are scattered by bone compared to skin. This is supported in the *in vivo* model of infrared light penetration through human hand versus human skin and triceps muscle, which was described above. Generalizing parameters of penetration through the human skull is complicated by the fact that the human skull has multiple bones, and their width varies from 6.5–18 mm, depending on the gender and age (33). The penetration of low power infrared light through isolated human skull from cadaver has been examined (21). Jagdeo et al. found that only 8.3% of the infrared photonic energy from a 50 mW LED device emitting 830 nm light (continuous wave – Omnilux) penetrated human parietal bone. The penetration of human frontal bone was less at 4.4% (21). Jagdeo et al. (21) estimated the power density reaching the cerebral cortex in their cadaver model was 3 mW/cm^2^, which equates to a fluence of 0.0064 J/cm^2^. This is 1/140th of the minimum thought to be necessary for ideal photobiomodulation (4). Hence, their work supports the premise that low power infrared light emitters do not deliver significant fluence through human skull.

**Figure 4:**
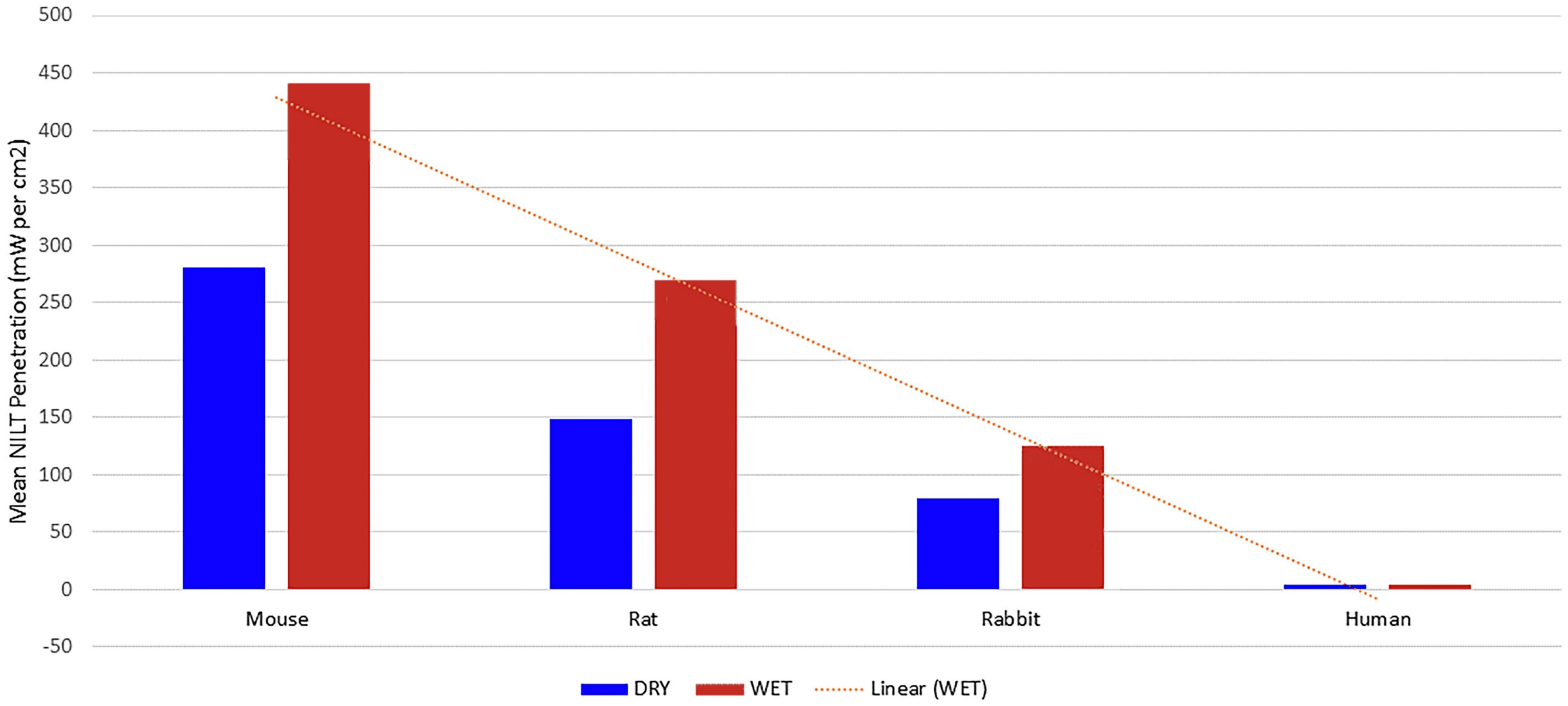
NIR light penetration through skull of three animal species and human cadaver. Line of regression shown as dotted line [Adapted from data provided in Lapchak et al. (34) and based on a graph presented in Henderson and Morries (7)].

Lapchak et al. (34) provided further evidence for this premise. They analyzed the penetration of NIR light through the skull of three representative animal species commonly used in NIR photobiomodulation studies, as well as samples of human skull (Figure 4). Using a dual wavelength laser (800 nm and 970 nm), they applied 700 mW to one surface of each skull in both the wet and dry state and measured the light penetration. Mouse skull measuring 0.44 mm thick, permitted 40.1% of the NIR light applied to one skull surface to penetrate to the other side. Rat skull measuring 0.83 mm thick, permitted only 21.2% of NIR light to penetrate the skull. Rabbit skull measuring 2.11 mm thick, blocked 88.6% of the impinging NIR light (only 11.4% of energy penetrated). Two different points were examined in the case of human skulls – the bregma and the parietal bone. The thickness of the human skull was measured and found to vary between 5.9–7.2 mm. The amount of incident NIR light which penetrated these two different spots in the human skull was only 4.2%. Lapchak et al. (34) calculated the incident power density based on this model to be 700 mW/cm^2^, while the power density dropped to 29 mW/cm^2^ or 0.029 J/s/cm^2^ on the undersurface of the human skull.

Recently, Castaño-Castaño et al. (35) have added additional data to the understanding of how infrared light penetrates, or fails to penetrate, the bone of the skull. This group examined the penetration of multiple wavelengths through 25 samples of frontal skull bone. They used laser diode devices tuned to specific wavelengths and delivering somewhat different, albeit low, power (405 nm–0.027 W; 532 nm–0.081 W; 655 nm–0.0577 W; 780 nm–0.1138 W; 810 nm–0.0607 W; 830 nm–0.1762 W; 1,064 nm–0.066 W) to the surface of the frontal bone and measured the amount of light penetrating the bone with an optical meter with a range of 10 nW to 2 mW. The penetration of key wavelengths was less than ½ of 1% (810 nm–0.2%; 830–0.22%; 980 nm–0.34%; 1,064 nm–0.5%). This study represents an important characterization of the differences of absorption and scatter for different wavelengths in bone (35).

### Penetration of heterogeneous tissues

Delivering infrared light energy to target tissues involves penetrating heterogeneous tissues. While studies of isolated bone or skin provide guidance, these studies ignore the scattering effects of the interface between these (and other) tissues. As stated above, the scattering coefficient of bone is approximately 22.9, while that of skin is 46.0. As a result, approximately 69% of infrared light energy is lost due to scatter alone prior to reaching the brain. However, additional scattering and reflectance which cannot be accurately calculated occurs at the interface between scalp and skull. Further scatter occurs at the interface of skull/dura, dura/cerebrospinal fluid (CSF), CSF/pia, pia/CSF, and CSF/brain. Furthermore, the brain, itself, has a scattering coefficient of 24.2 (Table 1). These more complex tissue configurations have been studied.

Anders’ group performed a particularly clever experiment which warrants becoming the standard for assessing the fluence of clinical protocols. Using 400 μm fibers with isotropic detectors connected to separate channels of a multi-channel light meter, they developed a probe which could provide accurate data on fluence at almost any given point within the brain. The team used an 808 nm laser at 5 Watts delivered to scalp surface of non-fixed cadaver heads. The thin fiber probes were inserted to known depths within the cadaver brain and light energy readings were recorded. Notably, at the surface of the cortex immediately subjacent to the lens of the laser, energy was detected in the range of 0.0029–0.027 mW/cm^2^. At 3 cm into the brain, the energy ranged from 0.00054–0.00095 mW/cm^2^ (36). Notably, these values for a 5 W laser fall well below the fluence range required to activate mechanisms at the level of the mitochondria.

Recently, Morse et al. (29) performed an experiment that this author has very much wanted to conduct. They utilized unfixed cadaver heads to directly measure the penetration of infrared light into the human brain. By placing a light meter inside the brain at the depth of 4 cm, they were able to directly measure the amount of 750 nm or 940 nm light delivered. The authors applied 4 W of continuous wave light to the shaved scalp (eliminating the confounding effects of hair) in four unfixed cadaver heads. In the frontal region, 0.032–0.278 mW was detected at 4 cm. Thus, approximately 0.0008–0.007% of the incident light reached 4 cm through scalp, skull, dura, and brain. In terms of fluence, this equals 0.000032–0.000278 J/cm^2^/s and this exposure would have to be maintained for 0.8–7.8 h to reach the minimum fluence shown to have photobiomodulation effects at the level of the mitochondria. In the parietal region, fluence was somewhat higher at roughly 0.000134–0.000396 J/cm^2^/s. These data correlate with the data from the studies conducted by the late Dr. Morries and I (4). We showed that there was a 99.995% drop in energy from a 6 W LED emitter when the light passed through 3 cm of scalp, skull, and brain in a fresh sheep head model.

**Figure 5:**
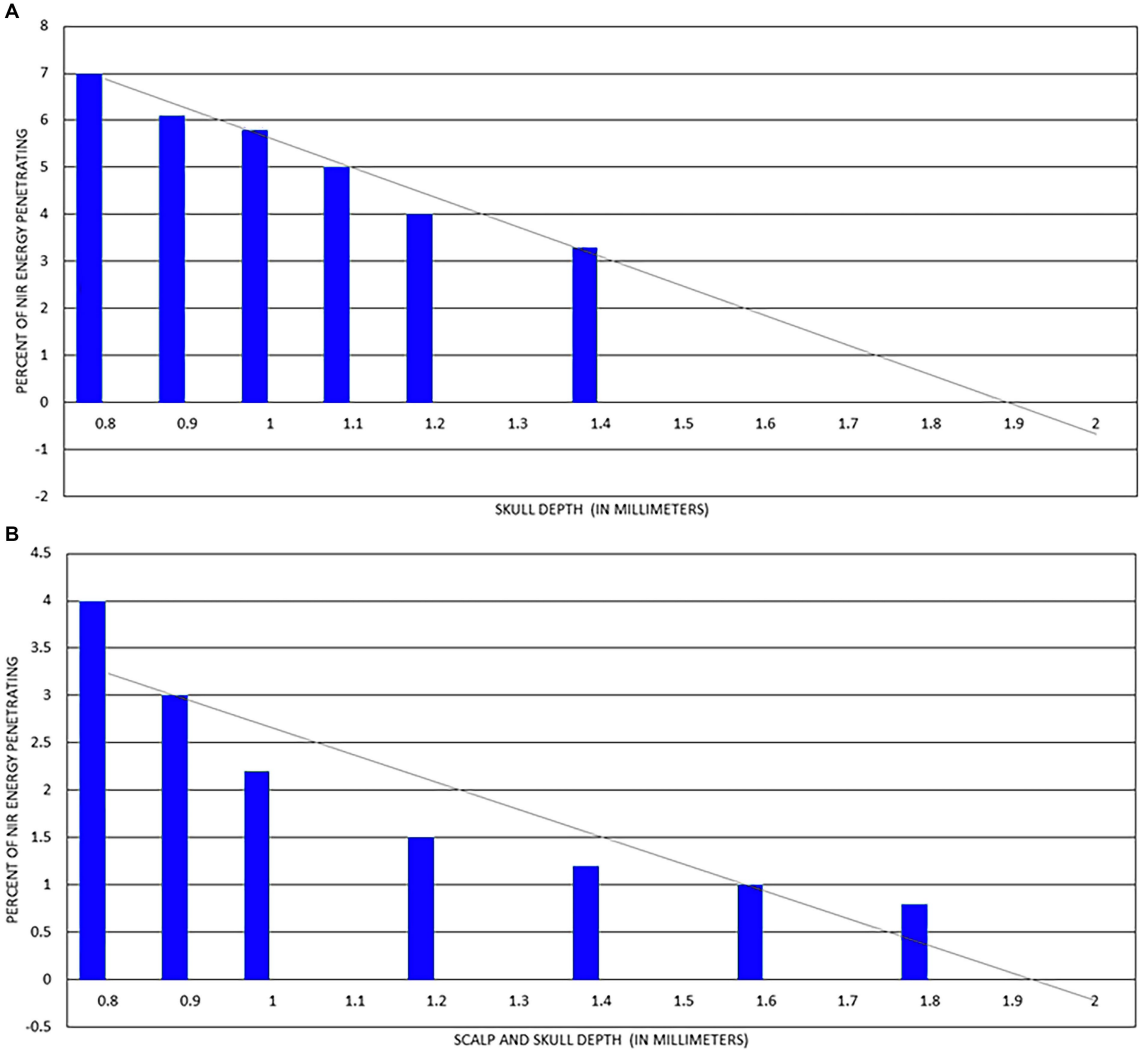
**(A)** Percent of 810 nm at 1 W NIR energy delivered to surface of skull which penetrates to internal surface of skull. Line of regression shown as grey line and indicates light at this power cannot penetrate more than 2 mm. **(B)** Percent of 810 nm at 1 W NIR energy delivered to surface of scalp which penetrates both scalp and skull to internal surface of skull. Line of regression shown as grey line and indicates light at this power cannot penetrate more than 2 mm [Adapted from data provided in Lychagov et al. (37) and based on a graph presented in Henderson and Morries (7)].

Lychagov et al. (37) examined penetration of infrared light through human cadaver scalp and skull using a 1 W 810 nm laser. They examined five different areas of the human skull – the vertex, frontal bone at the forehead, occipital bone at occiput, and temporal bone at the right and left temple. As shown in Figure 5, transmission of infrared light energy decreased with increased sample thickness. Indeed, if the loss of energy followed a linear regression, then it would penetrate no further than 2 mm through skull and less further through a combination of scalp and skull.

A few discrepancies in the field warrant elucidation. As previously elaborated, Jagdeo et al. (21) performed a similar study to that of Lychagov and colleagues above, using an 830 nm emitter delivering 50 mW and also examined penetration through human skull and overlying tissues at two sites on a cadaver human skull. They found that approximately 1.4% of 830 nm light energy penetrated approximately 10 mm of frontal skull and the overlying tissue in the cadaver model (21). Given the work of Lychagov et al. (37) and our own work showing 0% of the energy from a 50 mW 810 nm LED could penetrate 2 mm of human or sheep skin (not including skull) and only 9% of a 10 W 810 nm laser could penetrate the same thickness of skin (not including skull), the results of Jagdeo appear to be at least an order of magnitude out of alignment with others’ results.

Another odd inconsistency exists in the literature, Lampl et al. (18) conducted clinical efficacy studies on treating acute stroke using a GaAlA 808 nm diode laser device which delivered 70 mW and 268 J/cm^2^ to the scalp. The authors estimated this device delivered 10 mW/cm^2^ or 1.2 J/cm^2^ to the human cortical surface. This equates to less than ½ of 1% of the NIR energy delivered to the scalp reached the subjacent cortical surface. However, there is a point of confusion here. Oron et al. (14) used the same device from the same company in a study of infrared light penetration through mouse skull. The group precisely measured transmission of infrared light energy from the same GaAlA 808 nm diode laser through the parietal bone of fresh mouse skull. The laser delivered 21 mW to the outer surface of the skull for 2 min. and yielded 10 mW and 1.2 J/cm^2^ to the internal skull surface. Yet, the human clinical trials, utilizing the same device at the same settings and 2 min treatment duration, purported to deliver the same 1.2 J/cm^2^ to the cortical surface of human brain (18). This seems highly improbable given the much greater thickness of human scalp and skull through which the NIR energy had to penetrate in the clinical trials compared to the mere 2 mm thickness of mouse skull alone (14), as well as the results of Lapchak et al. (34) on the differences in infrared light penetration through mouse vs. human skull.

Lastly, NIR light transmission through living tissue likely is different than transmission through post-mortem tissue for several reasons (7). First, protein cross-linking begins soon after death and progresses. Second, changes in and loss of interstitial fluid occur within hours of death. Third, the movement of blood cells through the circulation of dermis and deeper tissues *in vivo* causes scattering and refraction of NIR light. Fourth, the flow of blood is an additional source of scatter but provides the benefit of dispersing heat from the site of NIR application. This was explored in greater detail in our earlier work (7) and was briefly discussed in Morse et al. (29).

### What can simulations teach us?

Several computer simulations of how infrared light penetrates overlying tissues and into the brain have been studied. Fitzgerald et al. (38) presented a diffusion simulation computer model delivering 28 mW/cm^2^ to the scalp and they derived that at the center of the brain (10 mm skull +46 mm brain = 56 mm), the power density would be approximately 1.2 × 10^−11^ W/cm^2^ for 670 nm light and 1.4 × 10^−7^ W/cm^2^ for 1,064 nm light. These values will result in fluence values that fall far below the fluence range predicted to have biological benefit of activating photobiomodulation at the level of the mitochondria. Pitzschke et al. (39) utilized Monte Carlo simulations using a model of the brain which excluded the scalp (gray matter, white matter, cerebrospinal fluid, thalamus, pons, cerebellum, ventricles, and uniform skull bone). The authors further simplified the model by using a uniform scattering anisotropy of g = 0.9. However, the scattering coefficients of tissue ranges widely from μ_s_ = 46.0 for skin to 24.2 for brain (7). Also, the refractive indices for bone and brain tissue were set at a uniform 1.37, despite wide variation in refractive indices across the tissues involved in transcranial photobiomodulation. The authors modeled several approaches to delivering infrared light to the subthalamic nucleus, including shining light inside the oral cavity. The resulting fluence in the depth of the brain was 1 × 10^−7^ J/cm^2^/s, many orders of magnitude below the fluence shown to be effective at the level of the mitochondria (0.9 J/cm^2^ to 15 J/cm^2^). Roughly 2,778 h would be required to reach a fluence of 1 J/cm^2^. Cassano et al. (40) also modeled the penetration of infrared light in the human head. These authors used the same parameters as Pitzschke et al. (39). In these Monte Carlo simulations, various positions on the scalp and in the nasal cavity were compared. The authors found that delivering 10^8^ photons of 670 nm, 810 nm, 850 nm, 980 nm, or 1,064 nm light at the F3-F4 position (per 10–20 system for electroencephalography) resulted in 1 × 10^−4^ J/cm^2^ reaching the dorsolateral prefrontal cortex, while 1 × 10^−7^ J/cm^2^ reached the ventromedial prefrontal cortex. Hence, it would require between 2.7 and 2,778 h to deliver fluence in the range shown to activate photobiomodulation at the level of the mitochondria.

### The inconvenient truth about hair

**Figure 6:**
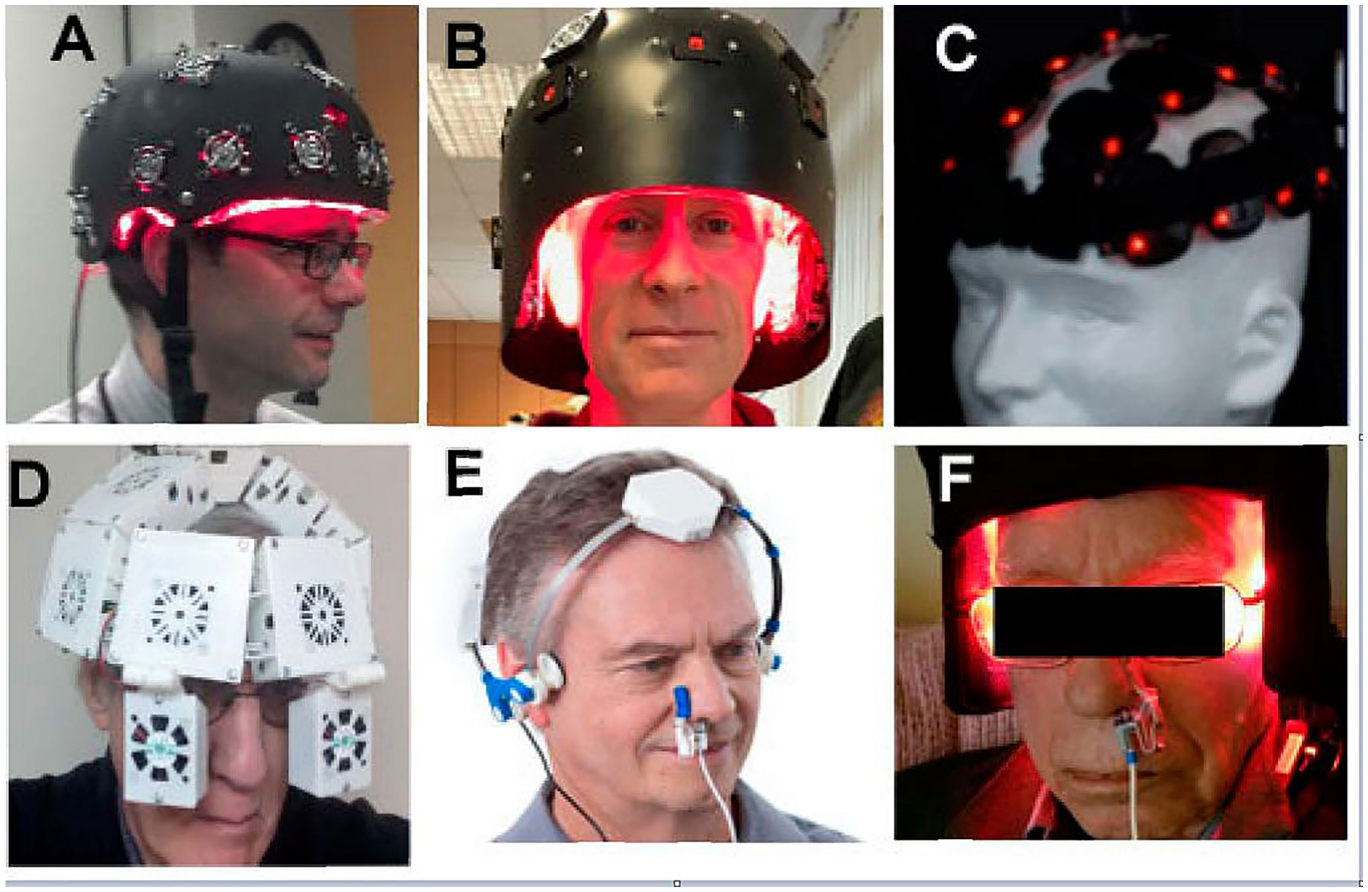
Numerous companies advertise LED devices which are placed on the head purportedly as a treatment for TBI and other neurological or psychiatric disorders **(A–F)** Typically, the devices are shown placed on top of the person’s hair. Since NIR is heavily attenuated by hair, this undoubtedly limits or eliminates any potential benefit from NIR irradiation [Originally published in Hamblin (26). Republished in Henderson and Morries (7). Used with permission].

Numerous commercial entities have begun advertising and selling infrared LED panels, helmets, and other apparatuses as “treatments” for brain disorders (Figure 6). While we do not dispute that case studies and small case series have shown clinical improvement using these low-power infrared light devices for patients with TBI, PTSD, and depression, one inconvenient truth that these images and the typical advertising ignore is that infrared light poorly penetrates hair.

**Figure 7:**
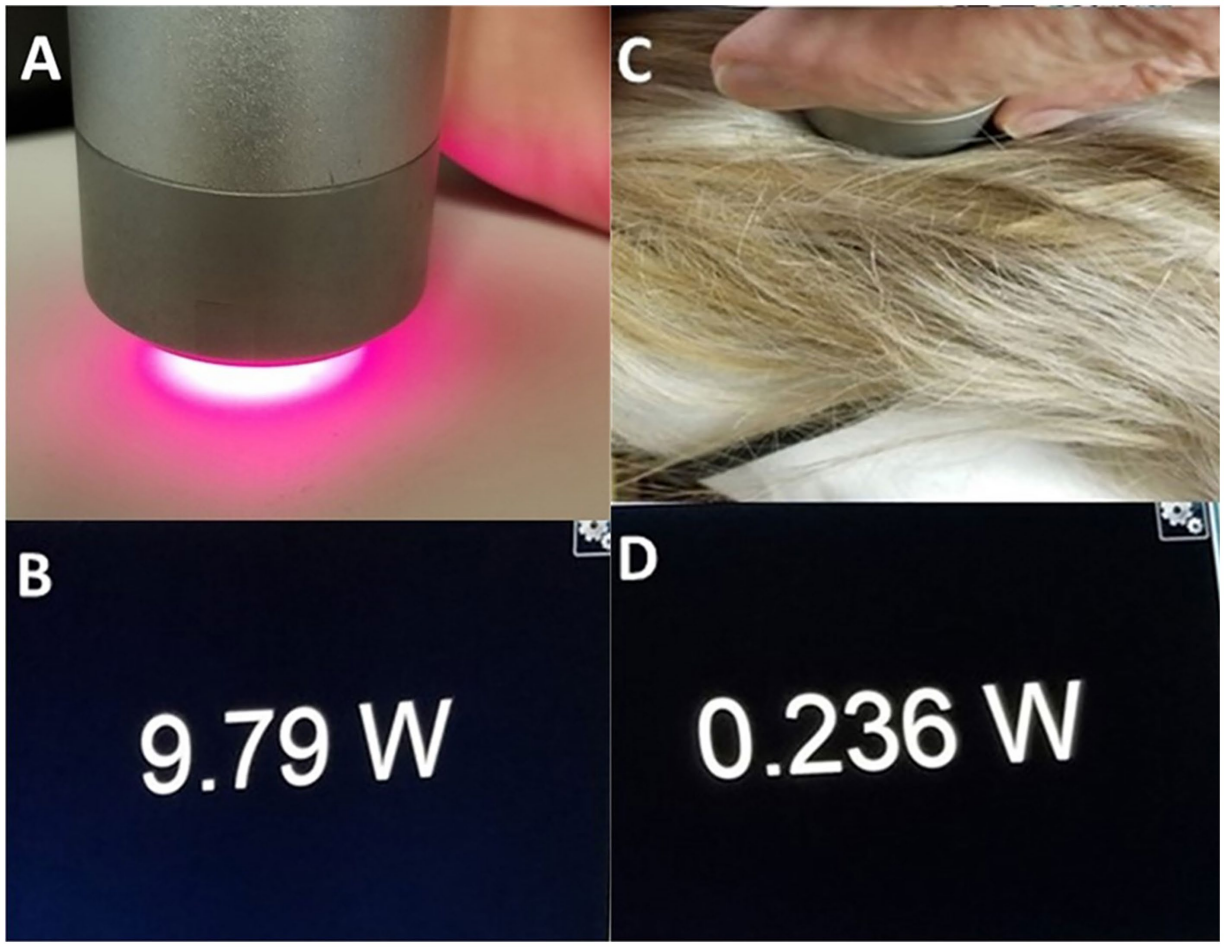
**(A)** Image of light meter display when a 10 W laser at 810 nm is placed 2 mm from a light meter. **(B)** The meter registers 9.79 W penetrating this distance of air. **(C)** A portion of hair approximately 2 mm in thickness is placed in between the NIR emitter and the light meter. **(D)** Image of meter display – only 0.236 W of NIR energy from the 10 W emitter can penetrate the hair to register on the meter [Adapted from Henderson and Morries (7). Used with permission].

It is magical thinking to believe or claim that placing a low-power NIR light source on top of a patient’s hair will result in treatment of the brain. We illustrated the potent blocking power of hair in a previous experiment (7). Figure 7 illustrates this simple, but powerful, demonstration of the ability of hair to absorb and block infrared light energy. While a 10 W laser placed 2 mm from a light meter delivered 9.79 W of energy to the meter, when a 2 mm mat of human hair was placed in between the laser head and the meter surface, over 97% of the infrared light energy was absorbed or scattered. When applied to a 0.5 W LED, the amount of energy that penetrates human hair is only 0.015 W. As we detail herein, scalp and skull also absorb, refract, reflect or otherwise block infrared light. The amount of light from a 0.5 W LED that can reach the brain becomes questionably small.

**Figure 8:**
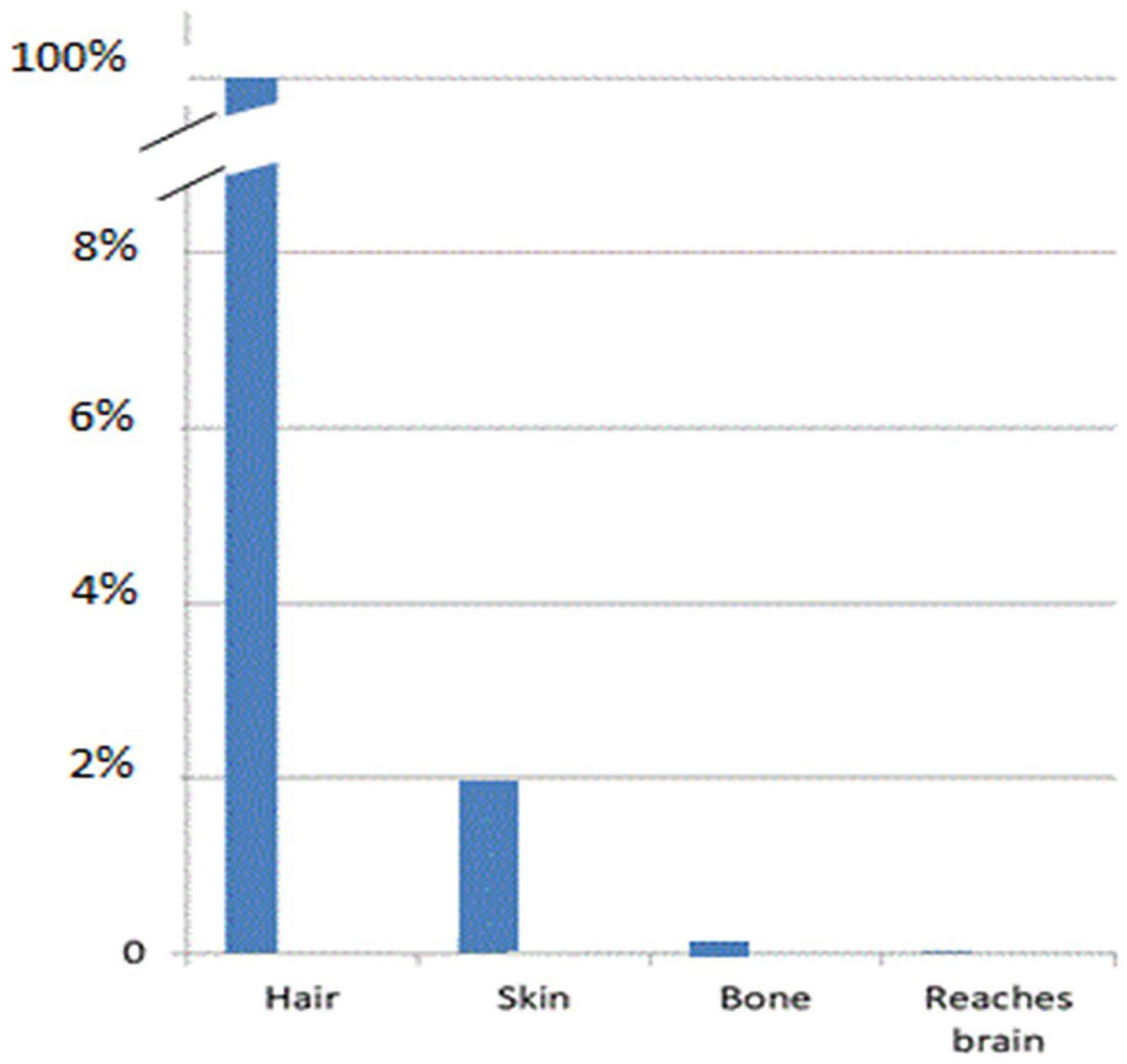
Graphic representation of the drop in light penetration at each layer of tissue (hair, scalp, skull, brain) that intervene between the surface application of infrared light energy and the target neurons within the brain.

When LED-based therapy devices, as shown in Figure 6, are placed over hair, very little NIR energy reaches the scalp, let alone the brain. If 98% of the energy from a 0.5 W LED is absorbed by hair (7), 91% of the remaining light is scattered by human scalp (4, 29, 37), 24% of the remaining NIR energy is scattered by skull (23), and 24% of what remains after that is scattered by 1 cm of brain tissue (23), then, based on the physical properties of *scatter alone*, the claims of direct photobiomodulation benefits of LED-based devices become highly questionable (see Figure 8). Thus, clinical case series, such as Hipskind et al. (41) and others (42–45) raise logical questions as to what is actually occurring to result in the reported clinical improvements.

This situation is further complicated by the fact that the human cerebral cortex is not a flat surface as found in the rat or mouse. Much of the cortical surface lines the floors and walls of the cortical sulci and so it not immediately subjacent to the skull. In addition, cortex immediately subjacent to the skull is not necessarily the focus of brain injury. As I have previously described (7), our prior reviews of the neuroimaging research has shown that the most commonly injured cortical areas in TBI are the inferior frontal (orbitofrontal) cortex and the temporal poles (46). Similarly, reaching the temporal lobes entails penetration through multiple tissues and multiple interfaces. This has been extensively reviewed previously (7).

### Pragmatic issues in reaching the target tissue in the brain

**Figure 9:**
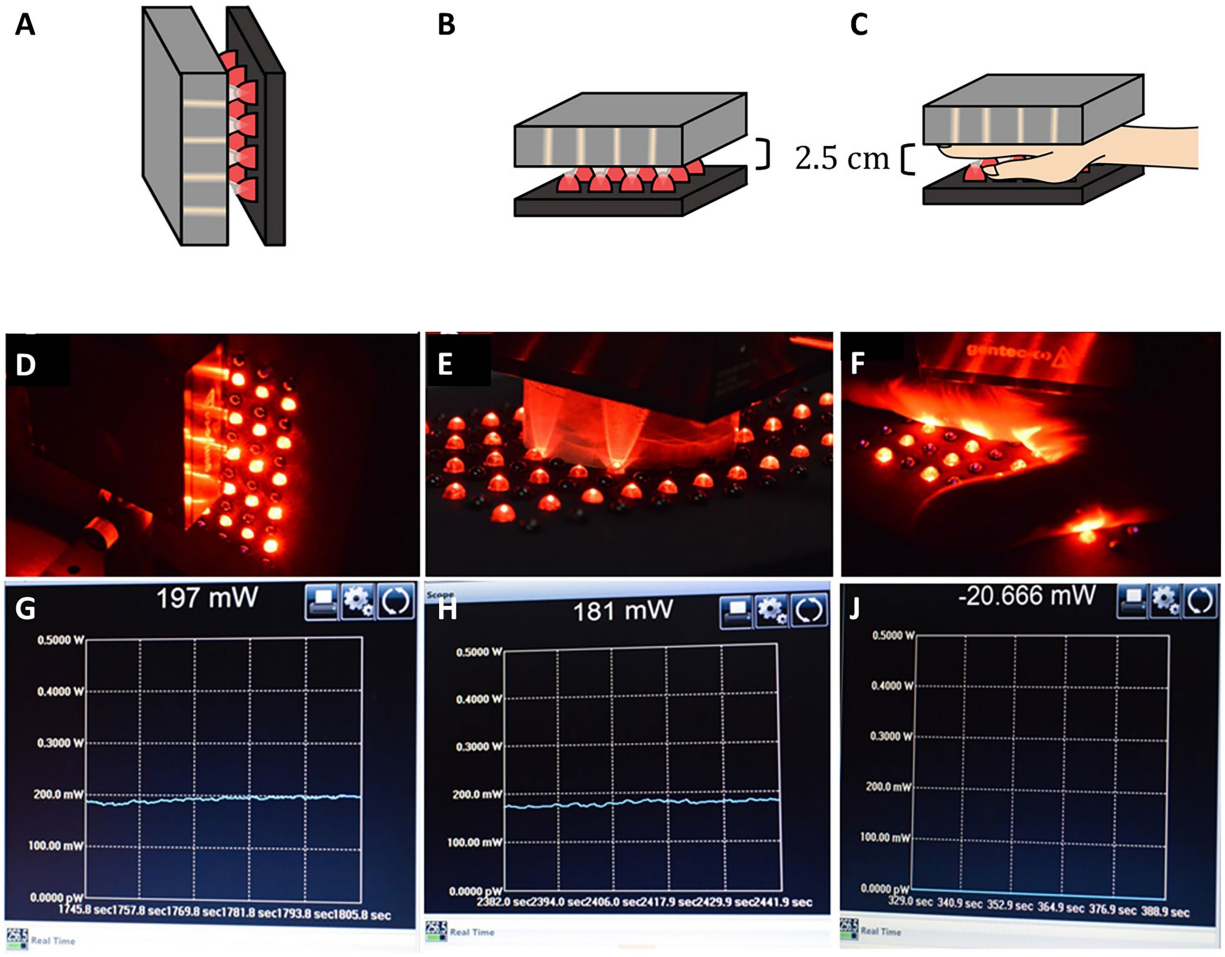
**(A,D)** An LED emitter at 830 nm with 500 mW output is placed in direct contact with a light meter. **(G)** The meter registers 197 mW, considerably less than 500 nW. **(B,E)** A cardboard cylinder measuring 2.5 cm in depth is placed between the LED emitter and the light meter. **(H)** The meter registers 181 mW is transmitted through 2. 5 cm of air. **(C,F)** A human hand measuring 2.5 cm in thickness is placed in between the LED emitter and the light meter. **(J)** The meter register no light energy being transmitted through the human hand over the course of 15 min [Adapted from Henderson and Morries (7). Used with permission].

The hypothesis assumed in the multiple clinical case series utilizing low-power LED devices described above in the discussion of scatter and penetration of NIR light through tissues can be summarized as follows, “Given enough time, even a low power LED emitter can transcranially deliver enough fluence for therapeutic benefit.” We previously studied the cumulative dose of NIR energy transmitted through 25 mm of heterogenous tissue over time using human hand as a model. This example was illustrated in our previous work (7). We had previously shown that only 4% of the energy from a 13.5 W emitter of 810 nm light penetrated 25 mm of living human hand (4). Here, we first determined the amount of photonic energy delivered by a low-power (0.5 W) LED emitting 830 nm infrared light. When placed in direct contact with a light meter, the meter registered an output of 197 mW. The LED emitter was then separated from the light meter by 2.5 cm of air using a cardboard cylinder. The meter registered a drop in photonic energy to 181 mW. Then the LED emitter was placed atop a living human hand which measured 2.5 cm in thickness and the light meter was placed under the hand. No photonic energy was detected to penetrate the human hand and reach the light meter. The meter was set to register the accumulation of photonic energy over the course of 15 min. No energy was detected during that time interval. This was illustrated in Figure 6.15 of our previous work (7). This simple illustration study demonstrates that if low-power infrared light emitters are not producing sufficient photonic energy to penetrate overlying tissue and reach the target tissue (or light meter in the illustrative example), then a protracted time interval will not lead to a slow accumulation of photonic energy at the target tissue (see Figure 9). Energy must be able to reach the target tissue to have a *direct effect*. Otherwise, something else is going on. We are not doing what we think we are doing with low-power infrared emitters and LLLT.

Our previous studies documenting how infrared energy penetrates 3 cm of sheep skull, brain and overlying skin and subcutaneous tissue are a close model of how infrared light penetrates in the clinical practice of transcranial treatment of the brain (4). Again, infrared energy emitted by devices generating less than 1 W could not be detected at the depth of 3 cm. A 99.995% drop in energy occurred when a 6 W LED system was used in this same model. In contrast, 0.14% of the NIR energy emitted by a 10 W 810/980 nm device could penetrate 3 cm of scalp, skull and brain tissue. In the same model, 1.26% of the energy from a continuous wave 810 nm emitter at 15 W was delivered to 3 cm depth. We have demonstrated that our multi-watt NIR data delivers an estimated 1.65–3.7 J/cm^2^ to a depth of 30 mm. As shown above, this is within the biologically meaningful fluence range (1, 2, 4, 6, 47) and is more than 100-fold greater than the fluence delivered by an LED system or by a low-power infrared light system according to the findings of the authors cited above (7, 18, 21, 37, 38, 48).

Our clinical experience supports, validates, and strengthens this position. Patients receiving 10–20 treatments of multi-watt infrared light, each lasting approximately 20–30 min, have experienced significant, and often, dramatic improvements (47, 48). The fluence of combined 810 and 980 nm light delivered during each of these treatments was, on average, 81 J/cm^2^/treatment. Correcting for forehead skin, skull, and 1 cm of brain tissue, this delivered a fluence of fluence of 0.41 J/cm^2^ to the neurons 1 cm below the cortical surface. Neurophysiological changes as shown by perfusion single photon emission computed tomography (SPECT) scans and consistent with the clinical improvements have been documented (5, 48). Moreover, these improvements persist for months and years (48). This stands in marked contrast to reported findings to date of transient benefits using LLLT and/or low-power LED devices (benefits begin to fade as soon as the treatment regimen is halted) (49–53).

Our first open-label clinical trial (47) included 10 subjects with mild TBI. Some had lost consciousness at the time of the injury, and all were many months post-injury. After a course of 10 treatments of multi-watt infrared light therapy using 810 nm and 980 nm simultaneous emission (20 treatments in four patients), all patients had significant clinical improvement with reduction or resolution of many of their symptoms, such as headache, irritability, depression, anxiety, memory problems, and poor concentration. These initial clinical observations have been extended to a clinical population of over 180 patients (48). Consistently, we see improvement in symptoms which occurs rapidly (under 10 weeks) and is persistent, in contrast to results obtained with LED devices. Physiological signs such as visual disturbance, impaired balance, and slowed reaction time also improve over the course of 10–20 multi-Watt infrared light treatments. This is not about the specific device used, rather the fluence at the skin/scalp surface that is sufficient to yield fluence to the neurons in the depths of the brain which can activate the mechanisms mediated by the mitochondria.

We have previously described three illustrative cases (48) of our extensive clinical experience. The cases ranged from mild TBI without loss of consciousness (LOC) to moderate TBI with brief LOC and severe TBI with prolonged coma. In each case, the patient experienced marked clinical improvement which was matched by neurophysiological improvement as evidenced by serial SPECT scans (48). Notably, we have followed our patients using bi-annual telephone interviews and the benefits have persisted for over eight years (48).

Recently, Rindner et al. (54) replicated our multi-watt open trial using a different wavelength (1,064 nm) and much lower power. Eleven patients were treated with 0.25 W/cm^2^ for 10 min using a continuous wave. They calculated a fluence of 120 J/cm^2^ delivered to the forehead over Brodmann area 10, which yields a fluence at the surface of the cortex of 0.45 J/cm^2^ based on the calculations I have provided herein. This fluence falls below the range established for direct PBM at the level of the mitochondria (0.9 J/cm^2^ to 15 J/cm^2^). Nonetheless, they report some subjective improvement and variable levels of improvement on standardized questionnaires. Unfortunately, they did not report on the persistence of these clinical changes.

The laboratory of Dr. Gonzalez-Lima also has contributed important work concerning biologically meaningful fluence. Using the longer wavelength of 1,064 nm, his group has demonstrated rapid and direct biological effects. Light in the wavelength of 1,064 nm appears to activate transmembrane mitochondrial complexes (complexes I, III, IV, and V) (55). Light of the 1,064 nm wavelength also demonstrates greater penetration (35, 56, 57).

In a double-blind study of healthy volunteers, single applications of 1,064 nm light improved memory and attention (58). Volunteers underwent baseline psychomotor vigilance and visual memory testing. Then, they received a single application of 1,064 nm light (3.4 W {area = 13.6 cm^2^}, power density 250 mW/cm^2^, fluence 60 J/cm^2^) to the right forehead, with the intent of targeting the right frontal cerebral cortex. When tested 2 weeks after the treatment, repeat testing revealed a slight, but significant decrease in reaction time (mean of 323 msec decreased to 317 msec) and a slight, but significant improvement in delayed visual memory (28.6 correct trials to 29.0 correct trials out of a possible 30). Applying scattering of skin (forehead) and skull based on Jacques (23) and Lapchak et al. (34), then the fluence delivered to the surface of the frontal cortex was approximately 1.26 J/cm^2^. This falls within the fluence range shown to be biologically active at the level of the mitochondria. Note that cortex in the sulci likely receive lower fluence.

Other studies using the same 1,064 nm, 3.4 W apparatus have demonstrated activation of mitochondrial electron transport chain (increased levels of activated terminal enzyme cytochrome oxidase) (59) and increased local perfusion (increased oxygenated hemoglobin) (59) in human volunteers after brief, 1-min exposures to 1,064 nm light. Longer, 8–11-min treatments using the same apparatus have been shown to induce electroencephalographic changes (60, 61) and improve depression (62). In summary, higher powered multi-watt emitters of infrared light have been shown to yield biologically verified and persistent changes in brain function. Penetration calculations show that fluence in the range shown to be biologically effective at the level of the mitochondria were achieved with these multi-watt studies. For the purposes of uniformity, I will employ the following computations for scatter and penetration: 2% of infrared light penetrates human hair (7), 50% penetrates human skin (forehead) (4, 23, 29), 9% penetrates human scalp (4, 29, 37), 4.2% penetrates human skull (34) and 76% penetrates 1 cm into human brain (23).

## Yet…LLLT is doing something

The penetration data presented above challenges the general notion that low-power infrared light, LLLT protocols, and/or low-power LED devices intended for home or clinic use can provide direct photobiomodulation to the brain. Despite the limitations of the penetrating ability of low-power infrared light energy from 0.5 W LED devices as detailed above, clinical benefits have been reported.

For example, Naeser et al. (52) reported on two patients with TBI who were treated with low-power infrared light (870 nm + 633 nm) delivered by 3 separate LED cluster heads. These LED cluster heads were positioned sequentially over the parietal region, bilateral forehead, high frontal region, temples, and the vertex. An LED cluster was also placed on the foot. Notably, the patients had only *transient clinical benefit* from this protocol. If the patients stopped treatment, then symptoms returned within two weeks. No neuroimaging was used to localize the lesion to which they were trying to direct infrared energy. Nor was there any neurophysiological measurement of change. Moreover, the delivery of infrared light energy to the foot had no direct effect on the brain. The authors (52) suggested a corollary to acupuncture points and hypothesized that the LLLT was increasing blood flow in the frontal lobe.

The same group (53) treated a small sample of patients (*N* = 11) with TBI using a similar LED-based device. They reported improvement in cognitive testing (Stroop Test and the California Verbal Learning Test) among a portion of the patients. Some of these improvements persisted. Scores on the Beck Depression Inventory decreased during active treatment, but reverted to pre-treatment levels after low-power treatment was stopped (53).

Another group used a custom-built helmet containing 360 LEDs which delivered 0.036 W/cm^2^ over a large surface area of the head (with hair) (63). Forty-three subjects with subacute TBI (within two weeks of injury) received either active treatment consisting of 3 sessions of 20-min exposure or similar sessions of sham. The authors estimated fluence delivered to scalp was 43 J/cm^2^ (ignoring the interference of hair). While some subjects in the active treatment group experienced decreased incidence of symptoms, such as headache, dizziness, and/or nausea, the study did not find a significant change in the primary outcome measure – scores on the Rivermead Post-Concussion Symptoms Questionnaire (64). Assuming the infrared light was applied over hair, the light actually reaching the scalp would be reduced by 97%, thus only 1.29 J/cm^2^ would reach the scalp (7). Applying the scattering coefficients described above (23), only 0.20 J/cm^2^ would reach 1 cm into the brain over 20 min. This falls below the above specified biologically active fluence.

As described above, Rindner et al. (54) treated patients with 0.25 W/cm^2^ delivered to the forehead. Subjects experienced some transient subjective improvement.

Saltmarche et al. (45) reported on the cognitive benefits of an LED panel device which included an intranasal LED. The device delivered 810 nm light at a fluence of 25 J/cm^2^ at the skin and was applied daily to four subjects over 12 weeks. The authors reported improvement in cognitive function at the conclusion of the treatment course; however, cognitive improvements diminished quickly after treatment was stopped (45).

Chao (65) used a similar device to assess the effects of low-level infrared light upon cerebral blood flow and measures of cognition in a group of patients with Alzheimer’s disease (65). She noted improvement in cognitive function and increased cerebral blood flow in the parietal cortex, although none of the values reached statistical significance. This study did not report on the long-term outcome after treatment was stopped. The LED panels over the parietal area of the skull in this study emitted 100 mW of 810 nm light. With a pulsing of 2 Hz, this yielded 60 J delivered per 20-min treatment. Applying the penetration calculations of Jagdeo et al. (21), 4.9 J of photonic energy are expected to penetrate the parietal skull bone alone. Applying the findings of Lapchak et al. (34), approximately 2.52 J of photonic energy would penetrate parietal scalp and skull to reach the surface of the cortex. Applying the findings of Lychagov et al. (37) described above, 0.3 J of photonic energy could be expected to penetrate scalp and skull in this protocol. Lastly, applying the scatter data of Jacques (23) and our previous observations of the interference caused by hair, then 0.0091 J/cm^2^ would reach the cortex in each 20-min session. This falls below the fluence necessary for direct PBM at the level of the mitochondria.

Cassano et al. (50) described a small open-label trial (*N* = 4) treating depression with an LED-based device. After six treatments over three weeks, Hamilton Depression Scale scores decreased from 19.8 ± 4.4 to 13 ± 5.4. The persistence of this benefit was not assessed. Subsequent studies of a different infrared emitter (823 nm, 28 LED delivering 33.2 mW/cm^2^ or 3.2 × 10–5 J/cm^2^/s to skin surface) were undertaken to assess the antidepressant benefit of infrared light (51) referred to as the ELATED-2 trial. With a 30-min treatment, this device delivered 0.059 J/cm^2^ at the skin surface on the forehead. Without correcting for hair (treatment applied to forehead), but applying the calculations and observations of Lychagov (37), Lapchak (34), Jagdeo (21) and others (4), only 0.0003 J/cm^2^ would be delivered to the cortex in each 30-min session. This falls below the fluence shown to induce PBM at the level of the mitochondria. In a sample of 21 subjects (10 PBM, 11 sham control), both self-rated scales and clinician ratings using standardized depression scales failed to show improvement, although the data trended toward significance in the ELATED-2 trial (51). In the ELATED-3 trial, the group (66) used a different LED infrared emitter (830 nm, unspecified numbers of LEDs delivering 54.8 mW/cm^2^ or 5.4 × 10^–5^ J/cm^2^/s at the skin surface of the forehead). Without correcting for hair, but applying the above cited calculations and observations of Lychagov (37), Lapchak (34), Jagdeo (21) and ourselves (4), only 0.0003 J/cm^2^ would be delivered to the cortex in each 20-min session. The ELATED-3 trial with 54 subjects showed no significant change in depression scores (self-rated or clinician-rated standardized depression scales).

Low-power infrared light has been applied to the clinical condition of autism. For example, Pallanti et al. (67) used an LED helmet to treat 21 children with autism. The helmet delivered 810 nm light at 100 mW pulsed at 10 Hz. The treatments were applied twice a day for 20 min over the course of 24 weeks. The total number of treatments was 240 (67). Two measures showed improvements. The first was the Childhood Autism Rating scale (CARS), which has a range of 15 to 60 and scores of less than 30 indicate a person is in the non-autistic range. A score greater than 37 indicates severe autism (68). Average scores in the sample dropped from 43 to 41. Similarly, a reduction in the score on the Montefiore Einstein Rigidity Scale (a measure of behavior rigidity) showed a decrease from 35 to 29. There was no control group, nor was there follow-up to see if these benefits were persistent (67). Applying the same mathematics to this example and assuming the device delivered 100 mW/cm^2^, then 0.0003 J/cm^2^ would be expected to reach 1 cm into the brain. A 20-min treatment would deliver a fluence at that depth of 0.006 J/cm^2^, which falls more than two orders of magnitude below the lowest range of biologically active fluence. Be mindful that this fluence is only at the surface of the brain. If the target is deeper tissue, such as the default mode network (7) (which is frequently implicated in autism), then greater photonic energy would be required to get into the range of fluence shown to have biological effects.

Another group examined the effects of infrared light in autism using a headband that delivered 850 nm light via LEDs. Treatments were applied twice a week for eight weeks with a total of 16 treatments (42). A total of 10 adults with autism participated in the study and a reduction on the Social Responsiveness Scale (SRS) was found. The SRS has a range of 0 to 195 with scores below 59 indicating a person is in the non-autistic range. A score greater than 75 indicates severe autism. The group who received treatment showed a 30-point reduction on the SRS. However, these changes were not persistent and faded after treatment was stopped (42). The most recent investigation into the treatment of autism involved 30 children treated with a headband LED device. The device delivered a dose of 300 mW of 850 nm light over the course of a treatment. Treatments were administered twice a week over eight weeks (69). The authors reported an improvement of 7.23 points on the CARS and favorable changes in EEG; however, the benefits of the treatment rapidly faded after treatment was stopped (70). Applying the same mathematics to this example and assuming the device delivered 300 mW/cm^2^ over the course of a single treatment, then 0.0009 J/cm^2^ would be expected to reach 1 cm into the brain, which again falls more than two orders of magnitude below the lowest range of biologically active fluence. The authors recognized that NIR energy is attenuated by the intervening tissues, noting:

“Future research is needed to clarify the optimal doses of tPBM, particularly for nonwhite patients who may require higher treatment doses due to greater light absorption losses by higher levels of skin melanin, which *reduces the penetration of light into brain tissues*.” (emphasis added; Frandkin et al., 2024).

Nonetheless, the authors fail to recognize that the amount of energy delivered to the brain is insufficient to induce PBM at the level of the mitochondria.

The late Dr. Morries and I previously reviewed several other aspects of the behavior in infrared light and its interactions with tissues, particularly the brain (7). For example, a given neuron in the brain does not experience the full power of all the LEDs in an LED device that is shining light on the head, but only receives, or potentially receives, energy from the LEDs to which it is directly subjacent. An LED over the frontal pole is not influencing a neuron in the parietal cortex. In addition, areas of the brain believed to be important in psychological and neurological disorders, such as the default mode network, are not necessarily accessible to superficial LEDs or low-power lasers (71). The default mode network is described as including the posterior inferior parietal cortex, retrosplenial cortex, medial prefrontal cortex, posterior cingulate cortex and neighboring precuneus, hippocampus, parahippocampal gyrus, and angular gyrus (72, 73). It is conceivable that infrared light from low power devices can reach the superficially located posterior inferior parietal cortex, superior portions of the medial prefrontal cortex, and the angular gyrus. However, key structures of the default mode network are much too deep for LED-derived NIR light energy to reach based on all of the evidence presented herein – specifically, the inferior aspects of the medial prefrontal cortex, deeper portions of the posterior cingulate cortex, parahippocampus, and hippocampus. As we discussed in that earlier work (7), penetration of energy from LEDs whether in the nasal cavity or on the surface of the skull (ignoring the interference of hair), cannot reach the deeper structures of the default mode network with sufficient fluence. The reader is referred to that earlier work for more details (7).

## Alternative explanations for the effects of low-power infrared devices

How then could low-power NIR energy be influencing brain function if it is not directly illuminating target neurons with sufficient fluence?

One potential explanation for the clinical benefits, albeit transitory, experienced by patients with brain injury or other neurological abnormalities treated with low-power NIR light is these changes were the result of a systemic effect. Braverman et al. (74) have demonstrated systemic effects of localized infrared irradiation on distant skin wounds. They inflicted full-thickness skin wounds on each forelimb of a rabbit model and then treated only one of them using a 632 nm/904 nm laser. The non-irradiated wound showed accelerated healing compared to wounds on untreated control animals (74). Rochkind et al. (75) demonstrated similar systemic effects in a bilateral wound model. Although only one side was treated, both sides showed accelerated healing. Moreover, they found that nerve crush injury also responded to systemic effects. They inflicted bilateral nerve crush injury, but applied irradiation only to the right sciatic nerve with HeNe laser (632 nm). Highly significant increases in action potentials were found not just in the right sciatic nerve, but in the untreated left sciatic nerve (compared to non-irradiated controls) (75). Similarly, Rodrigo et al. (76) utilized a rat model with three skin punch biopsy wounds to the back and then they irradiated the wound closest to the animal’s head with either 830 nm or 632 nm light at 50 mW. Curiously, the wound which showed the greatest histological evidence of healing was furthest from the point of light treatment.

Others have noted systemic effects. For example, Johnstone and colleagues (77–80) have repeatedly shown that irradiation of the back or abdomen of mice with either 670 nm or 810 nm NIR leads to a neuroprotective effect up to days later. Mice treated indirectly with NIR had reduced death of dopaminergic neurons after subsequent injection of the neurotoxin MPTP (78, 80). Guymer et al. (81) found that infrared light treatment of one retina for macular degeneration led to bilateral improvement in a series of 50 patients. Application of infrared light to the tail vein transcutaneously in a rat model can reduce pathology of ulcerative colitis [660 nm (82, 83)], accelerate peripheral nerve recovery [850 nm (84)], and reduce the severity of motor impairment after spinal cord injury [780 nm (85)]. Clinical trials of vascular photobiomodulation have been proposed (86).

The clinical work using low-level NIR light therapy supports the role of a systemic effect. For example, laser acupuncture to points on the trunk and limb of patients with depression lead to demonstrable perfusion changes in the default mode network as seen by functional MRI (87). In the clinical case series described by Naeser et al. (52), an LED cluster was applied to an acupuncture point on the foot. Similarly, in the later open-label trial the positioning of LED clusters was ascribed to acupuncture points (53). The authors postulated there was a benefit to cerebral perfusion by this technique and this is supported by the findings of Quah-Smith et al. (87). The mechanism of action by which acupuncture might improve cerebral perfusion is poorly understood and beyond the scope of this work. Nevertheless, acupuncture mechanisms would not have a *direct effect* on neurons subjacent to the LED cluster. Rather, acupuncture and low-power laser or LED treatment of acupuncture points must be exerting a systemic effect rather than a direct effect on intracranial structures.

Alternative mechanisms involving systemic elements may include metabolic modulators induced by low-level NIR light. Metabolic modulators have been shown to modulate the extent of excitotoxic secondary brain injury (88). Multiple lines of evidence suggest NIR light may increase nitric oxide levels (2, 4). Some authors suggest nitric oxide created at the site of NIR irradiation is carried throughout the body via the bloodstream leading to the beneficial effects of NIR phototherapy (41); however, nitric oxide is an extremely short-lived molecule (89). Alternatively, nitric oxide synthase activity might be induced, leading to overall increases in nitric oxide levels throughout the body (90, 91). A more reasonable hypothesis is that since NIR light has been demonstrated to induce certain growth factors, these may serve as systemic mediators. Irradiation of a small patch of skin on human volunteers resulted in the elevation of growth factors (92). Indeed, the plasma from treated subjects had growth promoting activity when added to the media of cells in culture (92). Treatment with 1,064 nm light to the oral mucosa led to increases in TGF-β in palatal wound fluid in human subjects (93). Lastly, NIR light has direct effects on inflammatory cytokines (91, 93, 94). These systemically acting agents – nitric oxide synthase, metabolic modulators, growth factors, and inflammatory cytokines – either separately or in some combination, may exert positive effects on the brain function.

The recent discovery of circulating cell-free mitochondria (95) raises an intriguing possibility that NIR phototherapy activates these mitochondria. Since wavelengths of 620–850 nm likely stimulate COX, while wavelengths near 1,064 may activate transmembrane mitochondrial complexes, free mitochondria within the blood of skin, scalp, and other tissues upon which NIR energy impinges may be activated. While it is not entirely clear that these cell-free mitochondria are capable of generating ATP via the electron transport chain, the molecular events initiated in mitochondria in response to certain wavelengths of NIR extend far beyond the production of ATP (see Figure 1). NIR energy may stimulate mitochondria (within cells or free-floating) to release signaling molecules, referred to a mitokines (96). It is well accepted that signals from the mitochondria, potentially in the form of mitokines, reach the nucleus of neurons and stimulate the upregulation of growth factors, such as BDNF. To the extent that these as-yet unidentified signaling molecules can circulate, it is possible that NIR energy from low-power LED devices, which have only sufficient energy to penetrate approximately 1–2 mm into the scalp, are actually stimulating mitokine release from mitochondria in the cells of scalp tissue, as well as cell-free circulating mitochondria. Hence, remote photobiomodulation results from the cumulative benefit of circulating factors upon the brain.

One way in which inroads have been made in identifying circulating molecules which may underlie the remote effects of NIR light is evidence for the induction of anti-inflammatory cytokines (97). For example, Zhevago et al. (94) demonstrated that a single NIR light treatment, as well as four daily treatments, led to elevated levels of circulating anti-inflammatory cytokines (94). The anti-inflammatory cytokine, tumor growth factor β-1 (TGFβ-1) levels increased 150%, while Interleuken10 (IL-10) levels increased 300%. In contrast, pro-inflammatory cytokine levels decreased. Tumor necrosis factor α (TNF-α) levels decreased 34-fold, while Interleuken-6 (IL-6) levels decreased 12-fold (94). Fukuda et al. (98) found similar results after NIR treatments in a mouse model (98). These systemic effects of NIR light therapy warrant further evaluation.

Lastly, my group has demonstrated remote photobiomodulation in humans by irradiating the ventral surface of the left forearm and inducing rapid increases in the alpha waves of the brain (99). This study explored the indirect effects of PBM on brain activity, specifically investigating whether applying NIR light to the forearm can induce changes in brain function. Nine author/participants received 1064 nm infrared light (9W X 6 minutes with 10 Hz pulsing = 27 J/cm^2^ per treatment interval) to the left forearm with quantitative electroencephalography (qEEG) recordings at baseline, and following irradiation of the left ventral forearm alternating with rest periods. Alpha power in the brain increased significantly following each irradiation session compared to rest periods, suggesting a potential indirect pathway for low-level PBM to affect brain activity. The study found that alpha power increased by 12% after the first treatment, 29% after the second, and 33% after the third, compared to the respective preceding rest periods.

These findings support the hypothesis that PBM applied to peripheral tissues can induce significant changes in brain activity, potentially through an unidentified systemic mediator. This challenges the traditional view that low-power PBM to the scalp is having a direct effect on the brain, as indirect PBM might be sufficient to elicit clinical benefits observed in treatments for conditions like traumatic brain injury, PTSD, and depression. Preliminary evidence showed that this effect could be blocked by placing a tourniquet on the upper aspect of the left arm prior to irradiating the left forearm with 1064 nm light (99).

## Implications for NIR spectroscopy

I am not stating that tiny amounts of NIR energy from low-power devices do not penetrate 1–3 cm through intervening tissues to reach the brain. Near-infrared spectroscopy (NIRS) is proof to the contrary (100). NIRS emitters deliver typically 0.1–0.5 W of incident NIR energy to the human scalp or skin (101). The optical pathlength (the distance that light travels through the tissues) is greater than the distance between NIR light source and the detector due to the scattering effects of the intervening tissues (102). Small amounts (<10%) of the light energy reaches the brain and is scattered by brain tissue as described above. Small numbers of photons travel 1–5 cm in the brain, pass through the skull and scalp again to be picked up by NIRS detectors (103). These detectors typically function in the nanowatt range (101, 104, 105). While photons of incident light do travel some distance into the brain, the fluence is far below that necessary to induce a direct photobiomodulatory effect. In mathematical terms, the amount of light which reaches the detector is 1×10^−9^ to 6 × 10^−11^ of the energy needed to activate PBM processes at the level of the mitochondria. Methods of modeling how “light travels through a highly scattering heterogeneous medium” (103) recognize that as the optical pathlength increases, the probability of photons being scattered greatly increases. These methods have included linearized solutions, Monte Carlo algorithms, and pragmatic modeling of each layer of tissue (106). Single channel functional NIR spectroscopy can assess global cerebral function, as needed in a critical care setting (102, 103, 107); however, multi-channel functional NIR spectroscopy is required to provide sufficient spatial resolution for brain mapping investigations (102, 103, 107). Efforts to improve the signal-to-noise ratio and control for the contribution of extracerebral tissues to the functional NIR signal are ongoing (108–110).

## Discussion and actionable recommendations

Infrared photobiomodulation embodies a remarkable and powerful tool for influencing mitochondrial function, inflammation, perfusion, and tissue repair mechanisms in the brain and other tissues. The challenge of translating success in the small animal model to the clinical situation in the human has been discussed at length. Currently, the actual mechanisms underlying the clinical improvement seen in patients with TBI, depression, and other neurological conditions as a result of infrared photobiomodulation remain unclear. This review of how infrared energy is attenuated as it passes through a variety of tissues strongly suggests the clinical benefit seen with low-power infrared light emitters may not be the result of the *direct effect* of the NIR energy on neurons. Rather, remote systemic effects may induce neurological changes which yield clinical improvement in the patient’s condition. Much in the way that remote photobiomodulation, systemic photobiomodulation, and vascular photobiomodulation invoke effects on distant tissue not directly exposed to the light, low power transcranial photobiomodulation may depend upon similar systemic effects and/or circulating molecules.

In contrast, our work and that of others (58, 59, 62) with multi-Watt NILT demonstrate that infrared light of sufficient power may *directly* activate the molecular mechanisms at the level of the mitochondria, which have been demonstrated in animal studies, within the neurons of the human brain. Using multi-Watt infrared light provides sufficient NIR energy to reach those neurons as we have demonstrated (4). If multi-Watt infrared light is, indeed, exerting a *direct effect* upon the neurons of the human brain, then this may explain why patients experience marked and persistent clinical improvement in their symptoms.

### Recommendations

Given the formidable evidence concerning the limitations of low-power infrared light penetrating tissues to directly reach the brain, certain recommendations can be made for further directions and research objectives. This is not intended to be a comprehensive list. The field of photobiomodulation is rapidly evolving.

1. Is the fluence range we specify as being necessary to induce mitochondria-related effects of photobiomodulation correct? The studies that support that data warrant repeating, best replicated in larger animal species (sheep, goat, dog) to assess if we correctly understand the fluence necessary at the level of the neuron. If this fluence range in the human or large animal model is actually 2–3 orders of magnitude lower, then low-power transcranial treatment might actually be doing what is claimed.
2. Once these rodent studies have been replicated in large animal models showing that stroke or TBI improve, BDNF is upregulated, synaptogenesis and dendritic arborization occur, etc., then the actual fluence in the brain of these large animal models should be accurately determined. The pioneering work of Tedford and Anders (36) and the recent study by Morse et al. (29) might serve as useful models to correctly determine the fluence of any given treatment once it has been shown to be effective in a large animal model.
3. Alternatively, studies of low-power photobiomodulation in large animals models of stroke or TBI may reveal little or no neurological benefit. If as contended in this article, low-power devices lack sufficient energy to penetrate scalp and skull and deliver fluence in the appropriate range, then dosing studies will reveal the power necessary at the scalp to deliver energy to the brain transcranially. Then Recommendation #2 is again relevant to understand the fluence delivered by multi-watt treatment modalities.
4. Large animal studies (sheep, goat, dog) are warranted to assess a model with scalp and skull thicknesses closer to that of human. Live animal studies are encouraged because of the differences in live and post-mortem tissues, the interactions at tissue interfaces, etc.
5. Large animal studies of commercially available low-power devices should be conducted to assess actual fluence delivered to the brain by these devices. This dovetails with Recommendations #1 & 2.
6. More comprehensive studies of the potential circulating signals released in vascular photobiomodulation, remote photobiomodulation, and systemic photobiomodulation are warranted. Barolet et al. (91) have offered numerous candidates, but careful experiments are needed to demonstrate which signal(s) are responsible in the human.
7. Comparative studies of candidate signal(s) in low-power transcranial photobiomodulation to determine what the signal(s) is/are and what is the overlap with signal(s) involved in systemic and remote photobiomodulation.

## Conclusion

The issue is a question of scale. Low-power infrared light therapy or LLLT likely lacks sufficient energy to penetrate the extent of overlying tissue in order to reach the human brain. The data presented herein from multiple studies is, indeed, overwhelming. These LED devices which are commercially available, particularly when placed over the patient’s hair, may be little more than placebo devices. As a result, the photobiomodulation field, as a whole, faces a serious concern. Claims made by those who do not understand NIR physics and light-tissue interactions can create confusion, doubt and distrust concerning NIR photobiomodulation. The field would do well to police itself. Claims should be supported by data and subject to scrutiny based on an understanding of light-tissue interactions. Direct photobiomodulation effects should not be claimed in the absence of data to support that sufficient fluence is delivered to the target tissue.

## Author contributions

TH: Conceptualization, Visualization, Writing – original draft, Writing – review & editing.
